# Relaxation Response Induces Temporal Transcriptome Changes in Energy Metabolism, Insulin Secretion and Inflammatory Pathways

**DOI:** 10.1371/journal.pone.0062817

**Published:** 2013-05-01

**Authors:** Manoj K. Bhasin, Jeffery A. Dusek, Bei-Hung Chang, Marie G. Joseph, John W. Denninger, Gregory L. Fricchione, Herbert Benson, Towia A. Libermann

**Affiliations:** 1 Benson-Henry Institute for Mind Body Medicine at Massachusetts General Hospital, Boston, Massachusetts, United States of America; 2 Department of Psychiatry, Massachusetts General Hospital, Harvard Medical School, Boston, Massachusetts, United States of America; 3 Department of Medicine, Massachusetts General Hospital, Harvard Medical School, Boston, Massachusetts, United States of America; 4 Department of Medicine, Division of Interdisciplinary Medicine and Biotechnology, Beth Israel Deaconess Medical Center, Harvard Medical School, Boston, Massachusetts, United States of America; 5 BIDMC Genomics and Proteomics Center, Beth Israel Deaconess Medical Center, Boston, Massachusetts, United States of America; 6 Institute for Health and Healing, Abbott Northwestern Hospital, Minneapolis, Minnesota, United States of America; 7 VA Boston Healthcare System, Boston, Massachusetts, United States of America; 8 Department of Health Policy and Management, Boston University School of Public Health, Boston, Massachusetts, United States of America; University of Texas Health Science Center at San Antonio, United States of America

## Abstract

The relaxation response (RR) is the counterpart of the stress response. Millennia-old practices evoking the RR include meditation, yoga and repetitive prayer. Although RR elicitation is an effective therapeutic intervention that counteracts the adverse clinical effects of stress in disorders including hypertension, anxiety, insomnia and aging, the underlying molecular mechanisms that explain these clinical benefits remain undetermined. To assess rapid time-dependent (temporal) genomic changes during one session of RR practice among healthy practitioners with years of RR practice and also in novices before and after 8 weeks of RR training, we measured the transcriptome in peripheral blood prior to, immediately after, and 15 minutes after listening to an RR-eliciting or a health education CD. Both short-term and long-term practitioners evoked significant temporal gene expression changes with greater significance in the latter as compared to novices. RR practice enhanced expression of genes associated with energy metabolism, mitochondrial function, insulin secretion and telomere maintenance, and reduced expression of genes linked to inflammatory response and stress-related pathways. Interactive network analyses of RR-affected pathways identified mitochondrial ATP synthase and insulin (INS) as top upregulated critical molecules (focus hubs) and NF-κB pathway genes as top downregulated focus hubs. Our results for the first time indicate that RR elicitation, particularly after long-term practice, may evoke its downstream health benefits by improving mitochondrial energy production and utilization and thus promoting mitochondrial resiliency through upregulation of ATPase and insulin function. Mitochondrial resiliency might also be promoted by RR-induced downregulation of NF-κB-associated upstream and downstream targets that mitigates stress.

## Introduction

The relaxation response (RR) is a physiological and psychological state opposite to the stress or fight-or-flight response [1], [2], [3]. Results from rigorous research studies indicate the ability of various mind-body interventions to reduce chronic stress and enhance wellness through induction of the RR [1], [4]. Several studies also reported that elicitation of the RR is an effective therapeutic intervention to counteract the adverse clinical effects of stress in disorders that include: hypertension [5]; anxiety [6], [7]; insomnia [8], [9]; diabetes [10]; rheumatoid arthritis [11]; and aging [12], [13].

The RR is elicited when an individual focuses on a word, sound, phrase, repetitive prayer, or movement, and disregards everyday thoughts [2]. These two steps break the train of everyday thinking. Millennia-old mind-body approaches that elicit the RR include: various forms of meditation (e.g., mindfulness meditation and transcendental meditation); different practices of yoga (e.g., Vipassana and Kundalini); Tai Chi; Qi Gong; progressive muscle relaxation; biofeedback; and breathing exercises [14]. Elicitation of the RR is associated with coordinated biochemical changes, characterized by decreased oxygen consumption [15], carbon dioxide elimination, blood pressure, heart and respiratory rate [16], [17], and norepinephrine responsivity [18], as well as increased heart rate variability [18], [19], and alterations in cortical and subcortical brain regions [20], [21].

Our previous study provided the first evidence that RR practice in healthy subjects at rest results in genomic expression alterations when comparing long-term RR practitioners to novices before and after their short-term RR training [22]. Specifically, sustained expression changes in genes significantly linked to oxidative phosphorylation, antigen processing and presentation, and apoptosis were identified in both short-term (N2) and long-term RR (M) practitioners at rest when compared to novices (N1) [22].

Regular daily practice of techniques that can be used to elicit the RR are often recommended for sustaining its beneficial effects. The immediate psychological and physiological effects from one session of RR-eliciting practice have been reported [1], [23]. We recently reported that these healthy subjects evoked psychobiological changes during one session of RR practice and that the psychological reactions correlate with biological changes only among long-term practitioners [23]. No study so far, however, has examined the acute changes in gene expression within one session of RR-eliciting practice and the impact of the length of previous practices on these immediate effects. In this current study, we determined the rapid temporal gene expression changes among these same study subjects using blood samples collected at 3 successive time points during a single RR practice session, which included listening to a 20-minute RR-eliciting CD. The healthy subjects served as their own controls since the identical temporal transcriptome analysis was performed on the novice subjects listening to a health education CD at baseline. The correlation between gene expression changes and biological changes within the session was also examined. It was hypothesized that in both long-term and short-term practitioners, one session of RR practice would evoke changes in gene expression that would be linked to a select set of biological pathways not observed in the naïve controls and that the changes would be more profound among long-term practitioners than those with short-term practice. Systems biology and interactive network analyses were employed to identify focus gene hubs of RR. Identification of these gene hubs, which are focal points or critical molecules in broad networks of interacting genes, could provide an empiric foundation for future investigations of genomic mechanisms of RR practices in specific clinical conditions. In addition, the investigation of the genomic expression changes that might occur during one session of RR practice will likely provide the scientific rationale for daily practice of RR elicitation, which is the common practice method recommended and followed by practitioners.

## Methods

### Study Design and Study Sample

Our study design is composed of both prospective and cross-sectional features (Fig. S1). The prospective aspect of the study involved enrolling 26 healthy subjects who had no prior RR-eliciting experience (Novices, N1) which served as their own controls. They then underwent 8 weeks of RR-eliciting training (Short-term Practitioners, N2). The cross-sectional aspect of the study involved enrolling another 26 healthy subjects who had significant prior experience of regular RR-eliciting practice for 4–20 years (Long-Term Practitioners, M) to be compared with novices either before or after their 8-week RR training. Study subjects were recruited from the Boston-area community using newspaper, on-line, and posted advertisements.

### Study Intervention

The long-term practitioners reported regularly practicing various RR-eliciting techniques including several types of meditation, Yoga, and repetitive prayer. They did not receive any intervention as part of this study. The novices received trainings in techniques to elicit the RR, which included 8 weekly individual training sessions from an experienced clinician in our research center. During the weekly session, subjects were guided through an RR sequence, including diaphragmatic breathing, body scan, mantra repetition, and mindfulness meditation, while passively ignoring intrusive thoughts. A 20-minute audio CD that guided listeners through this same sequence was given to the subjects to listen at home once a day [22].

### Data Collection

We collected blood samples and biological measures when study subjects attended morning laboratory sessions, during which M and N2 listened to a 20-minute RR-eliciting CD and N1 listened to a 20-minute health education CD (control). Blood samples for gene expression profiling were collected immediately prior to (T0), immediately after (T1) and 15 minutes after (T2) listening to the respective 20-minute CD (Fig. S1). Fractional exhaled nitric oxide (FeNO) samples were collected at the three time points in accordance with the American Thoracic Society guidelines [24]. The role of FeNO in explaining the physiological effects of RR, including reduction in blood pressure, has been hypothesized [25]. Our previous investigation provides preliminary evidence of the effect of RR in increasing FeNO levels [15]. FeNO is known to play a prominent role in vascular dilatation, which affects blood pressure [26], [27] and is also capable of influencing the character of immune responses [28].

### Data/Measurements Process Procedures

Total RNA was isolated from the peripheral blood mononuclear cells (PBMCs) in the blood samples as described previously [22]. Real-time exhaled FeNO was measured using a rapid response chemoluminescent Nitric Oxide Analyzer (NOA Model 280i, Sievers instruments; Boulder, CO), based on a previously described valid method [29], [30]. Before each laboratory session, two point calibrations were performed according to American Thoracic Society (ATS) recommendations [24] using a zero air filter and 45 parts per million nitrogen based calibration gas. Ambient NO levels were recorded before each measurement. On-line data were collected using Sievers NOAnalysis Software. Details are described by Dusek et al. [15].

### Transcriptional Profiling

For transcriptional profiling, the Affymetrix human genome high throughput arrays plates with 96 arrays (HT U133A), containing more than 22,000 transcripts, were used. Scanned array images were analyzed by dChip [31]. The raw probe level data were normalized using the smoothing-spline invariant set method, and the signal value for each transcript was summarized using the PM-only based signal modeling algorithm in which the signal value corresponds to the absolute level of expression of a transcript [31] (Details in Text S1).

### Data Analysis

**Figure 1:**
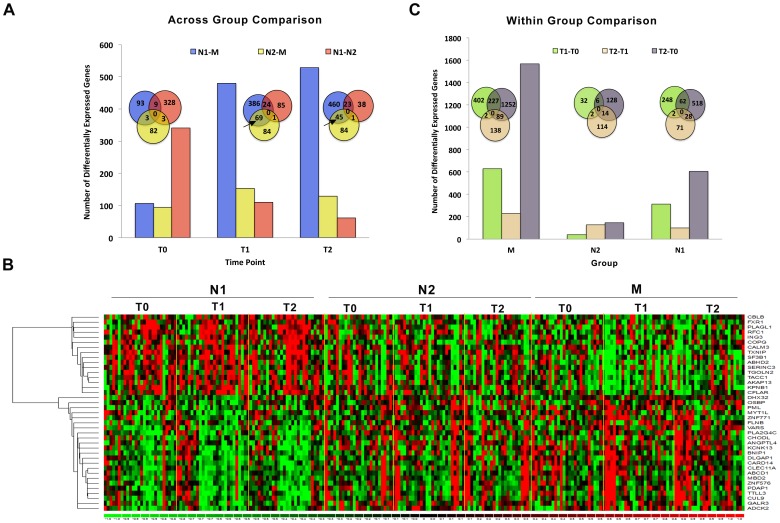
Individual gene-based differential expression analysis. A) Differentially expressed genes identified by 3 across-group comparisons (N1 vs. N2, N1 vs. M, and N2 vs. M) at T0, T1 and T2. Venn diagrams depict the overlap of genes identified by these 3 comparisons at each time point. B) Heat map of genes that were significantly differentially expressed comparing N1 vs. N2 and N1 vs. M at T1 and T2 (marked with arrow in Venn diagrams). Gene expression is shown with a pseudocolor scale (−1 to 1) with red color denoting increased and green color denoting decreased fold change in gene expression. The rows represent the genes and columns represent subjects in N1, N2 and M groups at T0, T1 and T2. C) Differentially expressed genes identified by 3 within-group comparisons at different time points (T0 vs. T1, T0 vs. T2 and T1 vs. T2). Venn diagrams depict the overlap of genes identified by the 3 comparisons within each group.

Data analysis for identifying RR affected genes and gene sets was conducted first based on individual genes then on biologically related gene sets using a hierarchy of bioinformatics techniques described below and outlined in Figure 1. We then conducted correlation analysis to examine whether the gene expression of RR affected gene sets are associated with the changes in biological measurement, FeNO.

#### A. Individual Gene Analysis

The individual gene analysis was implemented to identify differentially expressed genes based on intergroup (M, N1, N2) and intragroup (T0, T1, T2) comparisons using a random-variance t-test. The random-variance t-test is an improvement over the standard separate t-test as it permits sharing information among genes about within-class variation without assuming that all genes have the same variance. For the comparison of N1 vs. M or N2 vs. M at any time point the unpaired univariate t-test was used. The paired t-test was used for the time dependence within each group or comparison of N1 group with N2 group. Genes were considered statistically significant if their p value was less than 0.01. P-values for significance were computed based on 1,000 random permutations, at a nominal significance level of each univariate test of 0.01. To extract temporal expression patterns of individual genes that showed significant time and group differences, we then adopted the Self Organizing Map (SOM) clustering technique [32] (Details in Text S1). SOM allows grouping of gene expression patterns into an imposed structure in which adjacent clusters are related, thereby identifying sets of genes that follow certain expression patterns across different conditions.

#### B. Gene Ontology (GO) enrichment analysis

To identify the over-represented GO categories in the different gene expression patterns obtained from SOM clustering, we used the Biological Processes and Molecular Functions Enrichment Analysis available from the Database for Annotation, Visualization and Integrated Discovery (DAVID) [33]. The GO categories with *p-value*<0.05 were considered significant (Details in Text S1).

#### C. Gene Set Enrichment Analysis

The individual gene based analysis and SOM analyses are able to identify genes that depict large expression changes. However, subtle (but statistically significant) gene expression differences in biologically- and functionally-linked genes in response to RR might be missed using these two approaches. To overcome this analysis shortage we adopted the popular Gene Set Enrichment Analysis (GSEA) which was originally developed by Mootha et al for the purpose of identifying genes involved in oxidative phosphorylation that are coordinately downregulated in human diabetes [34], [35]. Gene Set Enrichment Analysis (GSEA) was used to determine whether a priori defined sets of genes showed statistically significant, concordant differences between 2 groups (N2 vs. N1, M vs. N2, and M vs. N1) or two time points (15 minutes vs. 35 minutes, 15 minutes vs. 50 minutes) in the context of known biological sets. GSEA is more powerful than conventional single-gene methods for studying the effects of interventions such as RR in which many genes each make subtle contributions (Details in Text S1). The enriched gene sets have nominal p-value (NPV) less than 5% and a False Discovery Rate (FDR) less than 25% after 500 random permutations. These criteria ensure that there is minimal chance of identifying false positives.

The genes from enriched pathways were merged into functional modules on the basis of overlap of significantly enriched genes using enrichment map plugin [36] in Cytoscape: An Open Source Platform for Complex Network Analysis and Visualization [37]. Genes with significant overlap (70% common genes) were considered neighbors and substitutable with each other. The patterns in significantly enrichment genesets from different comparisons (e.g. N1 vs. M, N1 vs. N2, 15 min vs. 35 min, 15 min vs. 50 min) were identified by developing a dotplots in lattice package. Details for pattern classification are described in the Text S1.

#### D. Pathways and Interactive network analysis

To gain an insight about pathways of the differentially expressed genes and gene sets we analyzed interactive networks and pathways for different patterns identified from GSEA analysis of differentially expressed genes using the commercial system biology oriented package Ingenuity Pathways Analysis (IPA 4.0) (http://www.ingenuity.com/). It calculates the P value using Fisher Exact test for each network and pathway according to the fit of user's data to the IPA database [38] (Details in Text S1).

#### E. Systems Biology analysis

To further identify pathways that are interconnected with known functions (e.g., protein-protein interactions and gene regulation interactions) genes of pathways from various patterns and generated an integrated network using known Protein-Protein, Protein-DNA and Protein-RNA interactions were selected. The interaction information was obtained using literature search, information from knowledge base databases such as MIPS, DIPS, HPRD and ingenuity systems [39], [40]. Networks were analyzed using the cyto-Hubba plug-in for Cytoscape 2.8 platform to identify network hubs and bottlenecks, which may represent the key regulatory nodes in the network [41]. The network consisting of the top 20 focus gene hubs was considered as the RR core signature network.

## Results

### RR leads to qualitative and quantitative temporal transcriptome changes: Individual Gene Analysis

We performed transcriptional profiling analysis on all samples using the Affymetrix HT Human Genome U133A Array Plate. After stringent quality control analysis [42], we conducted group comparisons on the normalized gene expression data using permutated univariate t-tests to identify differentially expressed genes. The across-group comparison identified the sets of genes that were significantly differentially expressed between groups at each time point (Fig. 1A). Both Short-term (N2) and Long-Term (M) practitioners evoked significant temporal gene expression changes as compared to novices (N1) during one session of RR elicitation. A larger number of genes were differentially expressed between M and N1 groups than between M and N2 or N2 and N1 at T1, right after listening to the CD, and at T2, 15 minutes after listening to the CD (Fig. 1A), indicating that long-term practitioners exhibit more pronounced transcriptional changes in response to RR elicitation. These results corroborate our previous observations that long term RR practitioners have more transcriptional changes as compared to short-term practitioners at rest [22].

We next determined which differentially expressed genes were common to individual pairwise comparisons between groups (e.g., M vs. N1). These common genes are shown as the intersecting areas of the Venn diagrams in Fig. 1A. There was a significant overlap of differentially expressed genes between M vs. N1 and M vs. N2 at both T1 and T2 (69 and 45 transcripts, respectively, marked by arrows in Fig. 1A). These overlapping transcripts, corresponding to 39 well-annotated unique genes (when duplicates and multiple transcripts from the same gene were removed), represent temporal expression changes across the different groups (N1, N2, M) and across the different time points (T0, T1, T2). The expression of these genes showed a gradually decreasing or increasing trend from N1 to N2 to M (Fig. 1B). Most of these genes are significantly linked to immune response, apoptosis and cell death based on Gene Ontology (GO) Enrichment analysis (P value<0.05) [33].

Similarly, the within-group comparison identified a number of genes that were differentially expressed across the three time points within each group (Fig. 1C). The long-term practitioners (M) demonstrated a rapid and more consistent response to RR elicitation in gene expression changes as indicated by a larger number of differentially expressed genes across the 3 time points (T1 vs. T0, T2 vs. T0, and T2 vs. T1; Fig. 1C) than both the short-term practitioners (N2) and novices (N1). In addition, a larger number of genes showed significant expression changes from T2 to T0 than from T1 to T0, indicating possible lag effects of RR elicitation in the M group. In comparison to the M group, the N2 group had lower numbers of consistently differentially expressed genes, which may be a reflection of a greater heterogeneity among short-term practitioners with regard to proficiency in RR elicitation.

### RR elicits distinct temporal patterns of differential gene expression: Self-Organizing Map (SOM) Analysis

**Figure 2:**
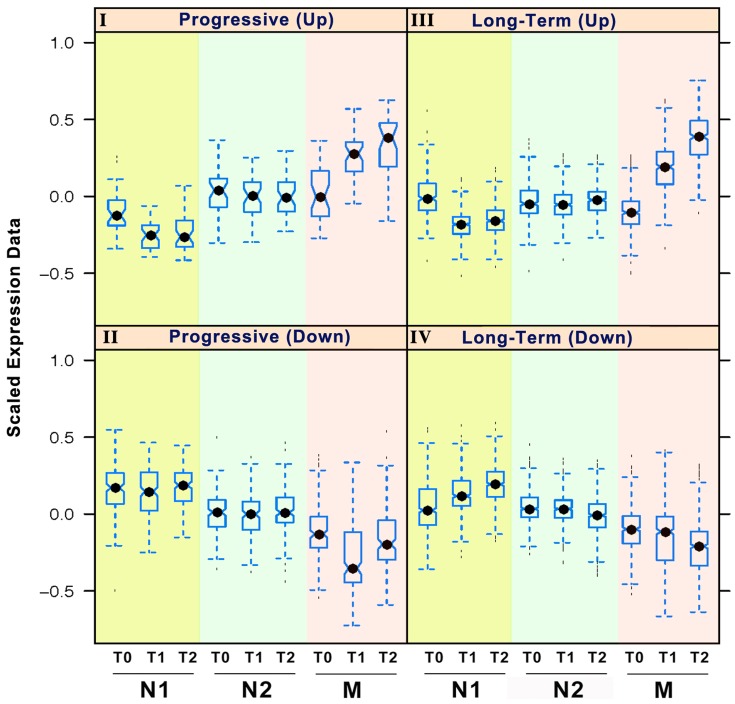
Temporal genomic expression patterns during one session of RR elicitation. Genes that were differentially expressed either across or within groups comparisons at different time points were used as the seed set of genes for Self-Organizing Map (SOM) analysis. These differentially expressed genes were partitioned to 18 separate maps according to Pearson correlation coefficient based distance metrics (Figure S2). Selected biologically interesting SOM maps were manually clustered into 4 biologically relevant categories based on the gene expression of N1, N2 and M groups at the 3 time points in one session of RR elicitation: Long-term Downregulation; Long-term Upregulation; Progressive Upregulation; and Progressive Downregulation. One representative pattern for each of these 4 biologically relevant categories is shown in the figure. The figure displays the box plot of the gene expression with X-axis representing time points and groups, and Y-axis representing scaled gene expression data from −1 to +1.

To identify temporal gene expression patterns, we performed SOM analysis on all of the differentially expressed genes identified by the individual gene analysis above [32]. Initially, differentially expressed genes were partitioned to 18 SOM patterns with different expression structures (Fig. S2). Based on their similarity in gene expression patterns we further merged these 18 patterns into four distinct categories of related patterns. These 4 categories reflect temporal gene expression changes (i.e., over minutes, from T0-T2) associated with RR elicitation in relation to length of previous practice (i.e., weeks to years): 1. “Progressive” Upregulation; 2. “Progressive” Downregulation; 3. “Long-term” Upregulation; and 4. “Long-term” Downregulation. Representative sets of patterns from Long-term and Progressive categories are shown in Fig. 2, which displays the box plot of the standardized gene expression values at each time point for the three groups. We defined a Progressive Upregulation pattern as genes with gradual increases in expression according to the length of prior RR practices — none (group N1), weeks-long (group N2), vs. years-long (group M) — and time trend within one RR session. In other words, the gene expression values were the lowest in N1, higher in N2, and the highest in M, especially at T1 and T2. In addition, the gene expression increased sequentially from T0 to T1 to T2 in M, but little change was observed in N1 or N2 across the three time points (Fig. 2, Panel I). GO enrichment analyses using DAVID [33] identified genes with Progressive Upregulation patterns to be significantly linked to regulation of cell differentiation, cell adhesion, cell communication and transport, hormone stimulus, blood pressure, cAMP, metabolic processes, biological oxidation, and oxidoreductase activity (Table S1).

In contrast, a distinct set of genes exhibited Progressive Downregulation patterns that had highest gene expression values in N1, lower in N2 and the lowest in M at all three time points. Furthermore, only the M group showed a decreasing time trend in gene expression from T0 to T1 (Fig. 2, Panel II). These genes are significantly linked to mRNA processing, intracellular protein transport, antigen processing and presentation, immune system, and primary metabolism (Table S1).

We defined Long-term Upregulation patterns as those for which gene expression levels were elevated in M compared to both N1 and N2, for which there were few gene expression differences between the two at all three time points. Only the M group showed higher expression across the three time points as compared to N1 and N2 (Fig. 2, Panel III). Long-term upregulated genes are involved in adenosine triphosphate (ATP) activity, protein binding, cell matrix adhesion, defense response, amine transport, response to stress, gap junction, and muscle cell differentiation (Table S1).

Similarly, we identified Long-term Downregulation patterns that contain genes with expression lower in M than both N1 and N2. In addition, only the M group exhibited a decreasing time trend across the 3 time points in gene expression. These genes are significantly associated with regulation of apoptosis, nuclear transport, metabolic processes, JAK-STAT cascade, T- and B-cell activation, regulation of cell cycle, insulin sensitivity, glucose transport, DNA replication, chemokine signaling, EGF signaling, and stress response (Table S1).

### RR progressively affected energy metabolism and inflammation pathways: Canonical pathways: Gene Set Enrichment Analysis (GSEA)

While identification of gene expression differences and gene expression patterns based on individual-gene analysis described above is able to reveal a subset of statistically significant changes in gene expression, subtle (but statistically significant) gene expression differences in biologically- and functionally-linked genes in response to RR might be missed in this analysis. Gene Set Enrichment Analysis (GSEA) is a statistical approach that identifies enrichment of sets of differentially expressed genes that share a common biological function or regulation [34], [35].

We performed GSEA to identify enrichment of the statistically significantly affected gene sets that are associated with various pathways by comparing two groups at each time point as well as comparing changes across time points within each group. A False Discovery Rate (FDR) <25% was used to indicate significant group difference and a p value of <0.05 was used to indicate significant time difference. As previously, we categorized enriched pathways into 4 patterns based on the number of time points with group differences in gene set expressions: Progressive Upregulation, Progressive Downregulation, Long-term Upregulation and Long-term Downregulation. The progressive patterns were further categorized into Progressive I and Progressive II patterns on the basis of the similarity between M and N2 groups on genomic expression changes in comparison to N1.

**Figure 3:**
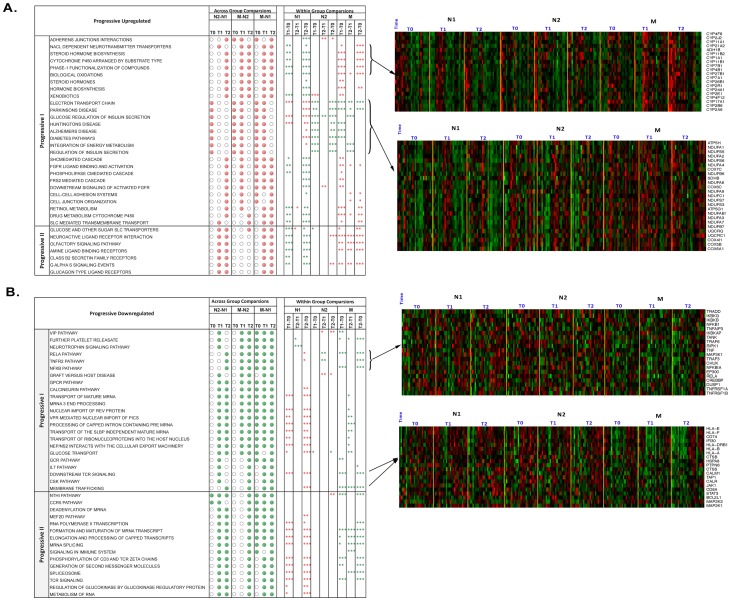
Significantly enriched pathways with progressive patterns identified using gene set enrichment analysis. A) Upregulated Pathways B) Downregulated Pathways. The solid dots indicate significantly affected pathways (False Discovery Rate <25%) identified from across group comparisons (N1 vs. N2, N1 vs. M and N2 vs. M) at a particular time point (T0, T1 and T2). The asterisks represent significance and directionality of enrichment (P value<0.09 *, P value<0.05 **, P value<0.01 ***) identified from within group comparisons at different time points (T0 vs. T1, T0 vs. T2, T1 vs. T2). The red and green color asterisks indicate up- and down-regulated enrichment of pathways respectively. The heatmaps depicting relative expression of selected genes from representative pathways are shown in panels on the right side. Gene expression is shown with a pseudo color scale (−3 to 3) with red and green colors denoting increased and decreased relative expression respectively. Pathways with progressive patterns were enriched (up- or down- regulated) in N2 and M groups with greater significance of enrichments in M group. Furthermore, increasing enrichment over time within one session of RR elicitation was observed in M group.

The Progressive I Upregulated pattern consisted of gene sets that were significantly enriched in both N2 and M subjects as compared to N1 subjects and with greater enrichments in M subjects at each time point (i.e., more time points with significant enrichments in M compared to N1 and N2) (Fig. 3A, solid dots indicating significant group differences). In addition to these across-group differences at each time point, most gene sets also showed significant changes across time points, particularly in the M group (Fig. 3A, asterisks indicating significant time difference). For example, gene sets for the cytochrome P450 (CYP450) family, steroid hormones, retinol metabolism, and cell adhesion pathways were upregulated in M and N2 with greater enrichment in M, based on both across- and within-group comparisons, as illustrated in the heatmap (Fig. 3A, Heatmap I). The CYP450 enzyme family is involved in the oxidative metabolism of a variety of compounds to regulate the production of reactive oxygen species that, in turn, regulate oxidative stress as well as many other signaling pathways and cellular functions [43].

Gene sets linked to energy metabolism (electron transport chain, integration of energy metabolism) and insulin secretion pathways were also upregulated in M compared to N1 and N2, as indicated by the significant group differences at T0 and T1 and the relatively higher gene expression values shown in the heatmap (Fig. 3A Heatmap II). Although there was a slight downregulation in gene expression across the three time points in M, the gene expression values remained higher in the M group than the N1 and N2 groups.

Similarly, GSEA identified Progressive Downregulated gene sets with Progressive I pattern based on both across and within group comparisons. These gene sets are linked to inflammatory processes (NF-κB, TNF R2, CCR5, IL-7, RELA) and T cell signaling pathways (Fig. 3B). The heatmaps clearly show the progressive downregulation across N1, N2 and M, as well as across time points in M (Fig. 3B, Heatmaps III and IV).

GSEA also identified pathways that had similar enrichments for M and N2 as compared to N1 where there was no significant difference between M and N2 at T0 and T1 (Fig. 3A, Progressive II). Only the M group, however, showed enrichment across time points in most of these pathways. We classified these pathways as Progressive II gene sets, for which both short-term and long-term practitioners (as compared to novices) had rapid enrichment within one session of RR practice. Progressive II Upregulated gene sets included pathways linked to glucose transport, neuroactive ligand receptor interaction and olfactory signaling, whereas downregulated gene sets were linked to immune response (CCR5, MEF2D, Phosphorylation of CD3 and TCR zeta chains, NTHI pathways) and mRNA preprocessing (maturation, metabolism, splicing and deadenylation) (Fig. 3B, Progressive II).

### Immune response and telomere maintenance related pathways are affected among Long-term RR practitioners: GSEA

**Figure 4:**
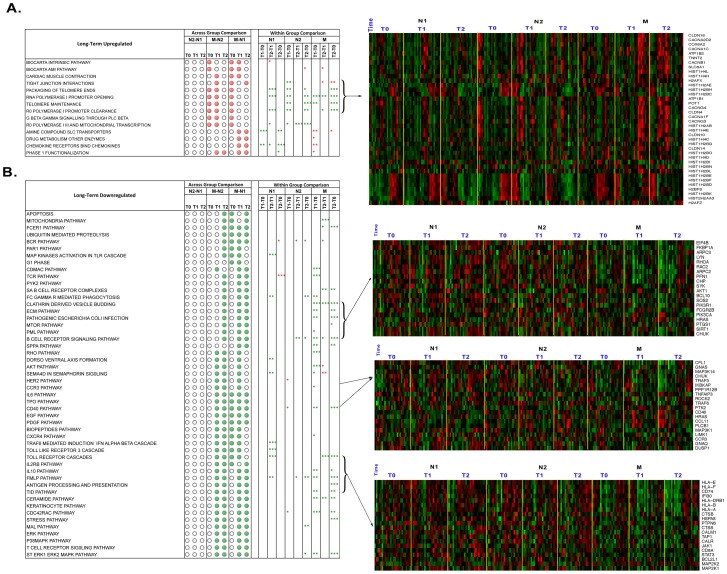
Significantly enriched pathways with long-term patterns identified using gene set enrichment analysis. A) Upregulated Pathways B) Downregulated Pathways. The solid dots indicate significantly affected pathways (False Discovery Rate <25%) identified from across group comparisons (N1 vs. N2, N1 vs. M and N2 vs. M) at a particular time point (T0, T1 and T2). The asterisks represent significance and directionality of enrichment (P value<0.09 *, P value<0.05 **, P value<0.01 ***) identified from within group comparisons at different time points (T0 vs. T1, T0 vs. T2, T1 vs. T2). The red and green color asterisks indicate up- and down-regulated enrichment of pathways respectively. The heatmaps depicting relative expression of selected genes from representative pathways are shown in panels on the right side. Gene expression is shown with a pseudo color scale (−3 to 3) with red and green colors denoting increased and decreased relative expression respectively. Pathways with long-term patterns were enriched (up- or down- regulated) only in M group. Furthermore, increasing enrichment over time within one session of RR elicitation was observed in M group.

GSEA identified pathways that were significantly upregulated in M subjects at 2 or 3 time points compared to both N1 and N2, for which there were no significant group differences. Some of these pathways, however, even though they were elevated in the M group as compared to N1 and N2, were downregulated within the M group from T0 to T2 (Fig. 4A, and Heatmap I). These pathways, classified as Long-term Upregulated pathways, were linked to telomere maintenance and cardiac muscle contraction (Fig. 4A). Likewise, GSEA detected several pathways that were significantly downregulated in the M group as compared to N1 or N2 groups at 2 or 3 time points. In addition, the M group showed a significant downregulation in gene expression from T0 to T2 (Fig. 4B, Heatmap II–IV). We classified these pathways as Long-term Downregulated pattern, which are significantly associated with immune response (antigen processing and presentation, TOLL receptor cascade, CXCR4, CCR3, IL6, CD40, TLR3, B cell receptor signaling, IL10 and IL2RB signaling, FC gamma mediated phagocytosis), cell cycle (apoptotic pathways) as well as stress-related pathways (stress pathway, P38 MAPK) (Fig. 4B). Indeed, the downregulation of immune and inflammatory response pathways and upregulation of energy production pathways are consistent findings in our data using multiple different analytic approaches.

### Upregulated Progressive changes induced by RR are linked to energy production in mitochondria: Systems Biology Analysis

To identify the key molecules — so-called focus gene hubs — affected by RR elicitation, we applied systems biology analysis to generate interactive gene networks. The interactive networks were generated from enriched genes of gene sets that are associated with RR practices identified by GSEA as described above. The networks were generated mainly on the basis of direct physical or biochemical protein-protein interactions, with a relatively small number of experimentally verified protein-DNA or protein-RNA interactions. The interaction information about the genes was obtained from public interaction databases or the Ingenuity commercial pathway analysis package [44], [45], [46], [47]. These interactive networks were further analyzed to identify network hubs using the bottleneck algorithm, which may represent the key nodes in the network. The focus hubs with higher degrees of connectivity are considered critical for maintenance of the networks, suggesting these might be critical in relaying beneficial effects of RR elicitation.

**Figure 5:**
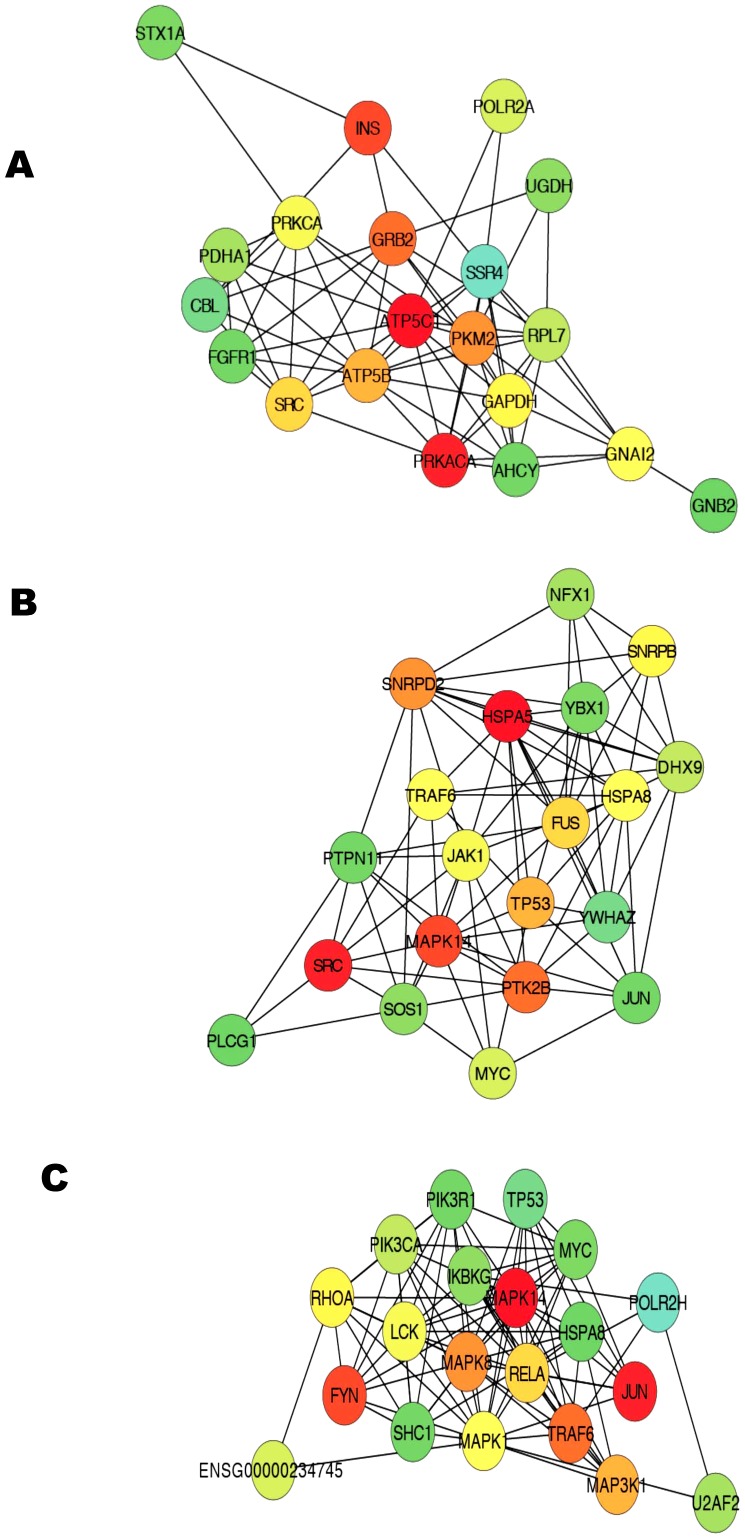
Interactive network and top focus gene hubs identified from significantly affected pathways. The figure represents the top focus genes. A) Progressive upregulated Pathways, B) Progressive downregulated Pathways, and C) Integrated network of Long-term and Progressive affected pathways. The top focus hubs were identified from complex interactive networks generated from pathways with progressive and long-term patterns. The focus gene hubs were identified using the bottleneck algorithm for identification of the most interactive molecules with tree like topological structure. The bottleneck algorithm ranks genes on the basis of significance level with smaller rank indicating increasing confidence. The pseudocolor scale from red to green represents the bottleneck ranks from 1 to 20.

The analysis on 27 upregulated pathways with the Progressive I pattern (Fig. 3A) generated a complex network that consists of genes from pathways related to energy production, metabolism, growth factors and glucose regulation (Fig. S3). Within this complex interactive network, we identified the top 20 bottleneck genes (focus hubs) with the highest number of molecular interactions with neighboring molecules. These focus hubs included the ATP synthase subunit gamma (ATP5C1), cAMP-dependent protein kinase (PRKACA) and insulin (INS) genes (Fig. 5A), all of which are linked to energy production and usage in mitochondria as well as glucose regulation [48].

### Upregulated Long-term changes induced by RR are linked to telomerase stability and maintenance: Systems Biology Analysis

The interactive network and focus hub identification analysis on 14 Long-term Upregulated pathways identified genes linked to DNA stability, recombination and repair (i.e., HIST1H2BC, CACNA1C, and CYC1) as the top focus genes. These genes play a critical role in telomere stability and maintenance (Fig. S4).

### Progressive and Long-term Downregulated gene expression changes induced by RR are linked to alteration of NF-κB-dependent pathways: Systems Biology Analysis

Interactive network analysis on the 23 downregulated pathways with Progressive I pattern generated a complex network that consists of genes from pathways related to inflammation, immune response, T cell signaling and mRNA processing. Within this interactive network we identified many of the top 20 focus hubs of these pathways to be related to NF-κB activity including MAPK14, MYC, PTPKB2, TP53, and TRAF6 (Fig. 5B).

The interactive network analysis on the 15 downregulated pathways with Progressive II pattern revealed a similar enrichment of NF-κB activity related focus molecules (e.g., RELA, TRAF6, MAPK14, MAPK11, TP53, MYC) (Fig. S5). The interactive network analysis on the Long-term Downregulated pathways also revealed the enrichment of NF-κB activity related molecules (e.g., MAPK1, MAPK3, JUN, SRC, TRAF6) (Fig. S6).

Finally, in an attempt to better understand the molecular mechanism of RR and to identify the most critical focus genes, we merged the Long-term and Progressive systems biology networks and investigated the focus hubs in this integrated network. The network of the top 20 focus hubs of this analysis clearly showed enrichment for NF-κB upstream and downstream target molecules (e.g., RELA, IKBKG, TRAF6, MAPK14, MAPK11, TP53, MYC) (FIG. 5C) and identified NF-κB associated molecules (e.g., MAPK14, HSPA5, PTK2B) as top focus hub genes, indicating the critical role of NF-κB in RR. These findings further support the notion that reduced NF-κB activity may be associated with RR elicitation.

### RR affected pathways are correlated with Fractional exhaled Nitric Oxide (FeNO) levels : Correlation Analysis

To evaluate whether changes of physiological or biological parameters acquired in the subjects before and after RR elicitation correlate with gene expression changes, we conducted correlation analysis on 10 selected pathways that were significantly affected by RR in Long-term or Progressive manner (Figs. 5, S6) using GSEA, an approach that is especially useful in identifying weak correlations and in hypothesis generation by using a set of biologically related genes instead of individual genes. The correlative analysis was performed on changes in gene expression and corresponding changes in FeNO levels in each group, as well as the measures at each time point.

We observed significant increases in FeNO level from T0 to T1 for M (p<0.01) and N2 (p<0.001), but non-significant increases in N1 (Table S2). The FeNO level remained elevated at T2 for M, but dropped significantly from T1 to T2 for N2 (p<0.0001) and N1 (p<0.01). The increases in FeNO from T0 to T1 in N2 were significantly (P value<0.05 and FDR<0.25) negatively correlated with Progressive Downregulated RELA and TNFR2 pathways (Table S3). For the M group, a similar pattern of negative correlation was also observed between changes in FeNO from T1 to T2 and the corresponding changes in gene expression of Progressive Downregulated RELA and long-term downregulated Antigen processing and presentation pathways (Table S3). In addition, changes in gene expression of Progressive Downregulated IL7 pathways and changes in FeNO levels from T0 to T2 were highly negatively correlated (p = 0.004, FDR = 0.007) in M group (Table S3). Furthermore, gene expressions of Long-term Downregulated Stress and Progressive Downregulated RELA pathways were significantly negatively correlated with FeNO levels at T2 in M group (Table S3). Finally, the MTOR pathway depicts a negative correlation with FeNO levels in both N2 and M groups, indicating a linkage between RR and MTOR activity, a pathway involved in regulating cellular hemostasis during stress [49], [50].

These negative correlations indicate that RR mediated downregulation of IL7, immune response and NF-κB/stress activation related pathways (RELA Pathways) are linked to increases in FeNO levels. FeNO is known for its role in lowering blood pressure through vascular dilation [26], [27] and is capable of influencing the character of immune responses. Our findings of the instant increases in FeNO after 20-minute RR elicitation among short-term (N2) and long-term (M) practitioners and the sustained increase 15 minutes later in the M group coupled with the significant correlation between FeNO changes and gene expression changes support the biological basis of the RR mediated pathways identified in the study.

## Discussion

Substantial research on mind-body interventions has established their ability to reduce chronic stress and enhance wellness through induction of the RR [2], [3], [4], [7], [14], [17], [18], [23]; however, little is known about the molecular mechanisms underlying RR-induced physiological changes. Previously, our group provided some of the first evidence that RR practice results in specific, lasting base-level gene expression changes that are opposite to transcriptional changes induced by chronic stress [22]. The study indicated that distinctive gene expression patterns associated with long- and short-term RR practices are sustained outside of RR-elicitation sessions. In contrast to our previous study [22], in the present study we interrogated the rapid and transient transcriptome changes (i.e., ‘temporal’ changes) during one session of RR practice among practitioners with years of practice (M) and novices before (N1) and after (N2) 8 weeks of RR training. We reasoned that temporal expression analysis across several time points would enable us to identify the immediate effects of one session of RR on gene expression and signaling and that these effects would differ among N1, N2 and M groups. Temporal analysis enables identification of genes that are affected by RR at multiple time points and reduces the likelihood of identifying false positives.

Analysis of the transcriptome data revealed that temporal modulation of gene expression occurred in both short- (N2) and long-term (M) practitioners as compared to novices (N1). Long-term RR practitioners exhibited more pronounced and consistent immediate gene expression changes as compared to short-term practitioners. Some genes were modified only in long-term practitioners (Long-term patterns), whereas others were modified in both short- and long-term practitioners with a greater intensity in the latter (Progressive patterns).

Importantly, this study demonstrates that during one session of RR practice rapid changes in gene expression (on the order of minutes) are induced that are linked to a select set of biological pathways among both long-term and short-term practitioners that might explain the health benefits of RR practices. These genes have been linked to pathways responsible for energy metabolism, electron transport chain, biological oxidation and insulin secretion. These pathways play central roles in mitochondrial energy mechanics, oxidative phosphorylation and cell aging [48], [51]. We hypothesized that upregulation of biological oxidation gene sets may enhance efficiency of oxidation-reduction reactions and thereby reduce oxidative stress.

The GSEA findings are further supported by the results from our systems biology analysis, which identified insulin (INS) and ATP synthase subunit gamma (ATP5C1) as top focus hubs. The mitochondrial ATP synthase is critical in regulating the production of adenosine triphosphate (ATP), which in turn is a key determinant for secretion of insulin from β-cells in response to glucose. Mutations in ATP synthase leading to its impaired signaling have been shown to induce oxidative stress and impaired insulin secretion in β-cells [51]. By upregulating ATP synthase — with its central role in mitochondrial energy mechanics, oxidative phosphorylation and cell aging — RR may act to buffer against cellular overactivation with overexpenditure of mitochondrial energy that results in excess reactive oxygen species production [52]. We thus postulate that upregulation of the ATP synthase pathway may play an important role in translating the beneficial effects of the RR.

Gene sets identified by GSEA as progressively downregulated by RR practices are linked to pathways that play critical roles in the inflammatory response, including those connected with the pro-inflammatory transcription factors NF-κB and RELA, and TNFR2, IL7 and TCR signaling. Systems biology analysis identified NF-κB associated molecules (e.g. MAPK14, HSPA5, PTK2B) as top focus hub genes. Downregulation of NF-κB inflammatory response gene sets may lead to reductions in oxidative stress, insulin resistance and apoptosis [53]. NF-κB has been identified as a potential bridge between psychosocial stress and oxidative cellular activation [54]. This supports our previous finding that RR significantly impacts the NF-κB cascade [22] at baseline in healthy subjects. A similar counter regulation of the NF-κB transcriptome was observed in a randomized controlled trial of a yogic mediation intervention in caregivers of dementia patients [55]. In addition to the RR, various other mind/body techniques have shown similar results such as the effect of cognitive-behavioral stress management on downregulation of the inflammatory cascade in patients with major illnesses [56]. Induction of NF-κB in PBMCs was observed in 17 of 19 volunteers upon psychosocial stress exposure and correlated with elevated catecholamine and cortisol levels. Likewise, the stress of awaiting breast biopsy has been found to activate NF-κB in women [57], and enhanced expression of stress-mediating MAPK14 was detected in PBMCs from graduate students under psychological stress [58]. In a vicious cycle, psychosocial stress can cause chronic mitochondrial oxidative stress that can lead to the metabolic syndrome (hypertension, obesity, insulin resistant diabetes mellitus, and hyperlipidemia) [59], [60]. This stress can lead to activation of NF-κB, which in turn can worsen oxidative stress and the metabolic syndrome.

Finally, NF-κB activation in PBMCs has been shown to correlate with peripheral levels of oxidative stress and can be reduced by therapeutic interventions that decrease oxidative stress [61]. Therefore, our finding that RR elicitation is associated with downregulation of the NF-κB node and its associated gene sets might be a key factor for explaining the clinical benefits of RR elicitation and provides a method for understanding the molecular mechanisms underlying the health benefits of RR through stress reduction.

Long-term RR practice, moreover, upregulated pathways associated with genomic stability such as telomere packing, telomere maintenance and tight junction interaction. Telomere dysfunction can cause disruption of mitochondrial regulators and cause mitochondrial compromise that ends in apoptosis [62]. Findings of several recent studies support our notion that mind/body interventions such as RR may enhance telomerase pathways. For example, a 3 month meditation intervention in 30 participants resulted in increased immune cell telomerase activity when compared to 30 matched control subjects [63]. In contrast, psychological stress has been linked to reduced telomerase activity, shortening of telomeres, and accelerated cell aging [64], [65]. Telomere length has been linked to insulin resistance and our findings of insulin signaling as a key target that is upregulated progressively as the time of RR practice increases corroborates this association [66].

Systems biology analysis identified histone (HIST1H2BC), calcium channel (CACNA1C) and cytochrome C (CYC1) among top focus hubs of the Long-term Upregulated pathways. HIST1H2BC is a core component of the nucleosome and is thereby essential to transcriptional regulation, DNA repair, DNA replication and chromosomal stability. Cytochrome C is an important member of the mitochondrial respiratory and energy production complex that again may provide an insight into the role of RR in mitochondrial energy efficiency. CACNA1C, a calcium channel gene, mediates the entry of calcium ions into excitable cells and is also involved in a variety of calcium-dependent processes, including muscle contraction, hormone and neurotransmitter release, gene expression, cell motility, cell division and cell death.

Similarly, pathway enrichment and systems biology analysis on long-term RR downregulated genes revealed associations with pathways involved in immune response (e.g. IL6, IL10, CCR3, antigen processing and presentation, TCR signaling), apoptosis (e.g. Apoptosis, Ceramide, PML) and stress response (e.g. stress pathway, MTOR). Psychological effects on PBMC gene expression associated with DNA repair mechanisms and immune response have been observed in women with postpartum depression, thus linking psychological stress to deregulated immune function and DNA repair that could be impacted by RR [67]. These results demonstrate the possible multi-level effects of RR in modulating immune and stress responses that counter stress-induced transcriptome changes.

In summary, we conducted the first study to employ advanced genomic analysis methodology and systems biology analysis to examine temporal transcriptional changes during one session of RR practice and found that RR practice induced upregulation of ATPase and insulin function. This suggests that RR elicitation may enhance mitochondrial energy production and utilization. At the same time RR induced downregulation of NF-κB-dependent pathways, with effects on upstream and downstream targets that may mitigate oxidative stress. These findings, while preliminary, suggest that RR practice, by promoting what might be called mitochondrial resiliency, may be important at the cellular level for the downstream health benefits associated with reducing psychosocial stress. Mitochondria have evolved the capacity to modulate specific anabolic and catabolic circuitries that control programmed cell death and autophagocytosis. They also confer an ability to sense the intracellular environment and help the cell adapt to a variety of stressors [68]. Mitochondria may be considered “master regulators of danger signaling” as well as important promoters of cellular resiliency and by extension perhaps resiliency of the organism itself [69].

The RR significantly affects multiple pathways through mitochondrial signaling that may promote cellular and systemic adaptive plasticity responses. In essence these adaptive responses become markers of what might be called **mitochondrial resiliency or mitochondrial reserve capacity**. The gene expression data indicate the RR specifically upregulates energy production of ATP through the ATP synthase electron transport complex. This might result in an enhanced mitochondrial reserve providing the capacity to meet the metabolic energy demands required to buffer against oxidative stress that emerges in many stress related diseases. Depending on variables such as genetic endowment and epigenetic interactions with micro- and macro-environmental circumstances, different mitochondria will have variable capacities to dampen the pathogenic effects of oxidative stress, and this has sometimes been referred to as **differential mitochondrial reserve capacity** [70]. When cells experience severe oxidative stress through increased cellular metabolic demands, there is a loss of mitochondrial reserve capacity contributing to a fall in mitochondrial resiliency, which may be a major contributor in disease vulnerability.

Our findings provide a framework for further deciphering the in-depth molecular pathways associated with the clinical benefits of the RR. To confirm this molecular mechanism of RR, validation of the results using secondary biochemical testing will be necessary.

## Supporting Information

Figure S1 Schematic view of temporal relaxation response study design and analysis plans. The transcriptome profiling was performed on peripheral blood mononuclear cells (PBMCs) collected immediately prior to (T0), immediately after (T1) and 15 minutes after (T2) listening to a 20-minute Education CD by the Novices (N1) or a 20-minute RR CD by the Short term practitioners (N2) and the Long term practitioners (M). The global transcriptome of PBMCs was profiled using HT_U133A arrays containing >22,000 transcripts. The transcriptome data were analyzed using high-level bioinformatics algorithms to identify differentially expressed transcripts, significantly affected pathways and systems biology networks that are related to RR elicitation. The expression patterns were generated from differentially expressed genes using Self- Organizing Maps (SOM) analysis. The results of the GSEA from all comparisons were classified to temporal patterns (e.g. Progressive, Long) by developing a Rlanguage script.
(PDF)

Figure S2 Temporal genomic expression patterns during one session of RR elicitation. Genes that were differentially expressed either across or within groups comparisons at different time point were used as seed sets of genes for Self-Organizing Map (SOM) analysis. These differentially expressed genes were partitioned to 18 separate maps according to Pearson correlation coefficient based distance metrics. Each pattern represents a set of genes that depict a similar expression pattern suggesting that they are biologically linked to a specific function. The figure displays the box plot of the gene expression with X-axis representing time points and groups, and Y-axis representing scaled gene expression data from −1 to +1. The patterns are merged into 10 expression categories on the basis of similarities in expression patterns.
(PDF)

Figure S3 Interactive Network of progressively (Progressive II) upregulated genes. The network was generated from genes of 27 progressively upregulated pathways (Progressive I) related to energy production, metabolism, growth factors and glucose regulation. The interaction information about the genes was obtained from public interaction databases or the commercial Ingenuity package. In a network each node represents a gene and an edge represents an interaction (e.g. protein-protein, protein-DNA or protein-RNA). The nodes with high degree of connectivity (Top 20) are highlighted in yellow color.
(PDF)

Figure S4 Interactive network and focus hubs of genes depicting Longterm Upregulation patterns. A) Interactive network, B) Top 20 focus genes. The interactive network and focus hub identification analysis was performed on genes from 14 Long-term Upregulated pathways linked to DNA stability, recombination and repair. In the network each node represents a gene and an edge represents an interaction. The focus gene hubs were identified using the bottleneck algorithm for identification of the most interactive molecules with a tree like topological structure. The bottleneck algorithm ranks genes on the basis of significance level with smaller rank indicating increasing confidence. The pseudocolor scale from red to green represents the bottleneck ranks from 1 to 20 (Fig. S4B).
(PDF)

Figure S5 Interactive network and focus hubs of genes depicting acute Progressive (Progressive II) Downregulation patterns. The interactive network and focus hub identification analysis was performed on genes from 15 Progressively Downregulated (Progressive I) pathways linked to mRNA processing and immune response. The focus gene hubs were identified using the bottleneck algorithm for identification of the most interactive molecules with a tree like topological structure. The bottleneck algorithm ranks genes on the basis of significance level with smaller rank indicating increasing confidence. The pseudocolor scale from red to green represent bottleneck ranks from 1 to 20.
(PDF)

Figure S6 Top focus gene hubs identified from Interactive networks of significantly affected Long-term Downregulated pathways. The figure represents the top 20 focus genes identified from complex interactive networks generated from pathways with Long-term Downregulated patterns. The focus gene hubs were identified and ranked using the bottleneck algorithm for identification of the most interactive molecules with a tree like topological structure. The pseudocolor scale from red to green represent bottleneck ranks from 1 to 20 (smaller rank indicating increasing confidence).
(PDF)

Table S1 Gene—ontology enrichment analysis of progressive and long—term expression patterns.
(PDF)

Table S2 FeNO levels during one session of RR elicitation.
(PDF)

Table S3 Correlation analysis of NO levels and Selected 10 pathways affected progressively or only in long term manner by RR (Bold). The correlation analysis was performed both by comparing FeNO and gene expression levels at particular time point (e.g. T0, T1, T2) as well as changes in gene expression and FeNO levels within a group. The significance of the correlation was determined on the basis of P value (P<0.05) and FDR (<25%). The positive and negative correlations between FeNO and gene expression levels are indicated by red and green color respectively.
(PDF)

Text S1 Supporting Information
(PDF)
