# Correction: Relaxation Response Induces Temporal Transcriptome Changes in Energy Metabolism, Insulin Secretion and Inflammatory Pathways

**DOI:** 10.1371/journal.pone.0172873

**Published:** 2017-02-21

**Authors:** Manoj K. Bhasin, Jeffery A. Dusek, Bei-Hung Chang, Marie G. Joseph, John W. Denninger, Gregory L. Fricchione, Herbert Benson, Towia A. Libermann

The Competing Interests statement is incorrect. The correct Competing Interests statement is: The following authors hold or have held positions at the Benson-Henry Institute for Mind Body Medicine at Massachusetts General Hospital, which is paid by patients and their insurers for running the SMART-3RP and related relaxation/mindfulness clinical programs, markets related products such as books, DVDs, CDs and the like, and holds a patent pending (PCT/US2012/049539 filed August 3, 2012) entitled "Quantitative Genomics of the Relaxation Response": MKB, JWD, GLF, HB, TAW.
