# Supplementary figures and images for: Relaxation Response Induces Temporal Transcriptome Changes in Energy Metabolism, Insulin Secretion and Inflammatory Pathways
Source: PLoS One. 2013 May 1;8(5):e62817. doi: 10.1371/journal.pone.0062817 (PMC3641112; doi:10.1371/journal.pone.0062817)

**Novices (N1)****Short-Term Practitioners (N2)****Long-Term Practitioners (M)****Pre-Visit (Week 0)****Post-Visit (Week 8)**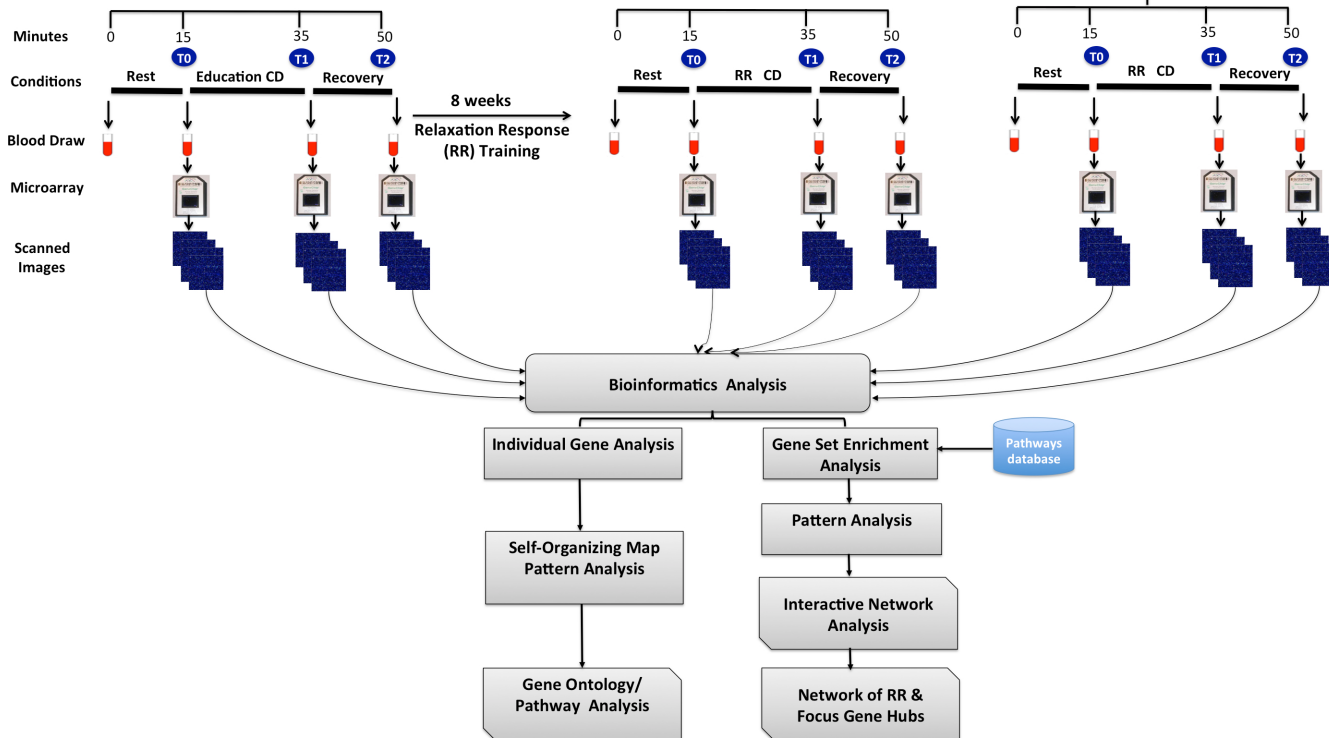

Supplement: Figure S1 — Schematic view of temporal relaxation response study design and analysis plans. The transcriptome profiling was performed on peripheral blood mononuclear cells (PBMCs) collected immediately prior to (T0), immediately after (T1) and 15 minutes after (T2) listening to a 20-minute Education CD by the Novices (N1) or a 20-minute RR CD by the Short term practitioners (N2) and the Long term practitioners (M). The global transcriptome of PBMCs was profiled using HT_U133A arrays containing >22,000 transcripts. The transcriptome data were analyzed using high-level bioinformatics algorithms to identify differentially expressed transcripts, significantly affected pathways and systems biology networks that are related to RR elicitation. The expression patterns were generated from differentially expressed genes using Self- Organizing Maps (SOM) analysis. The results of the GSEA from all comparisons were classified to temporal patterns (e.g. Progressive, Long) by developing a Rlanguage script. (PDF) [file pone.0062817.s001.pdf]

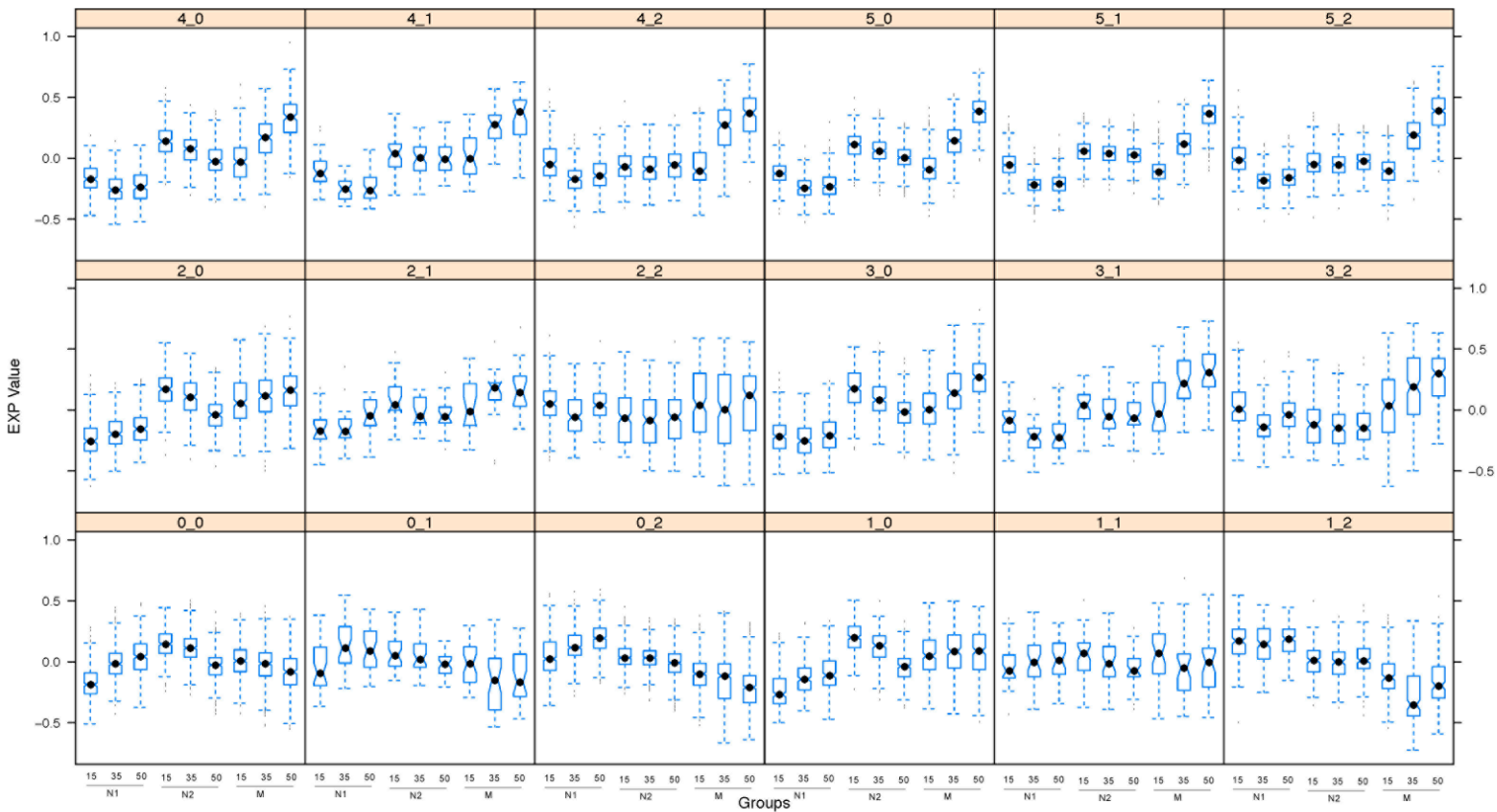

Supplement: Figure S2 — Temporal genomic expression patterns during one session of RR elicitation. Genes that were differentially expressed either across or within groups comparisons at different time point were used as seed sets of genes for Self-Organizing Map (SOM) analysis. These differentially expressed genes were partitioned to 18 separate maps according to Pearson correlation coefficient based distance metrics. Each pattern represents a set of genes that depict a similar expression pattern suggesting that they are biologically linked to a specific function. The figure displays the box plot of the gene expression with X-axis representing time points and groups, and Y-axis representing scaled gene expression data from −1 to +1. The patterns are merged into 10 expression categories on the basis of similarities in expression patterns. (PDF) [file pone.0062817.s002.pdf]

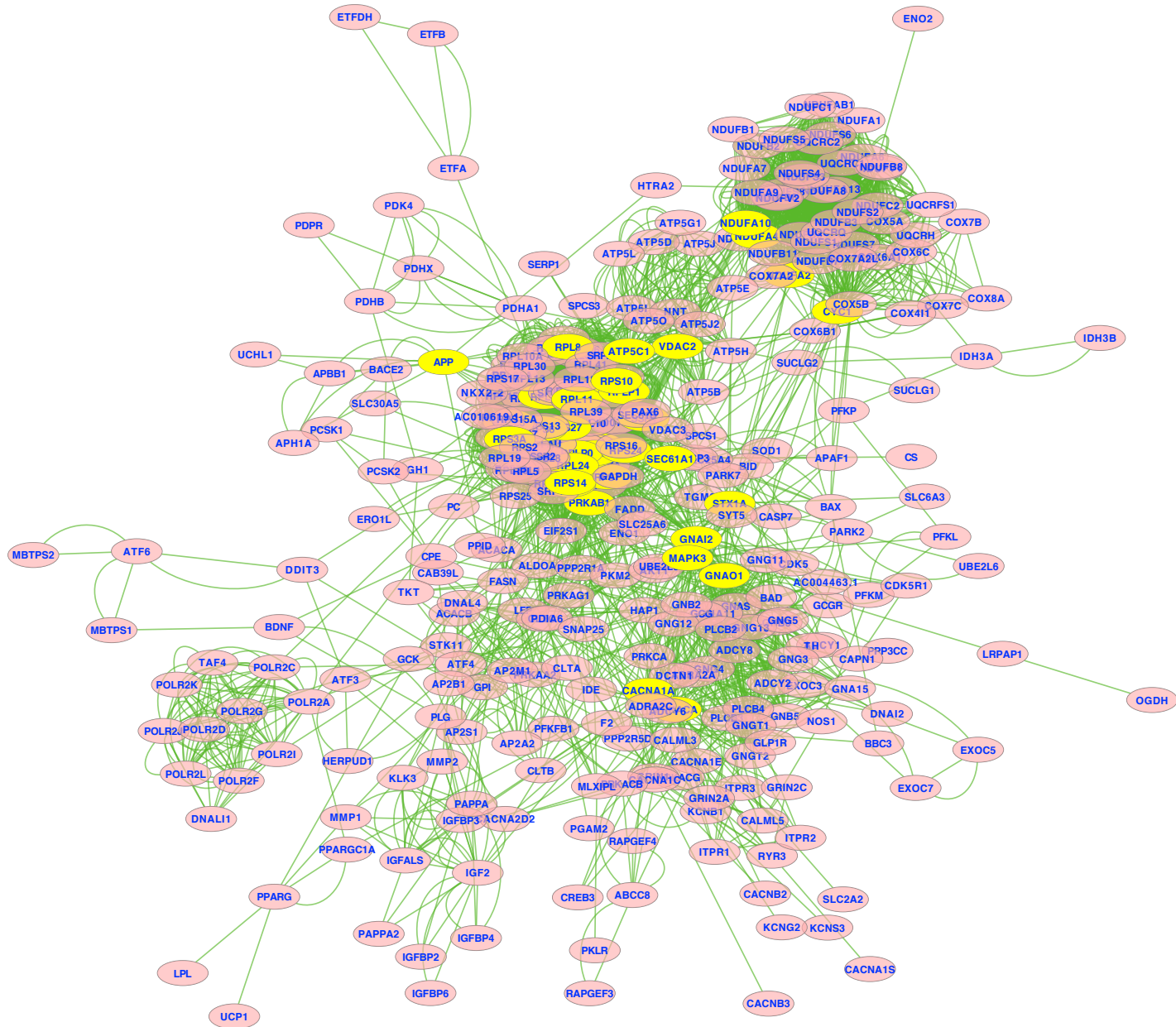

Supplement: Figure S3 — Interactive Network of progressively (Progressive II) upregulated genes. The network was generated from genes of 27 progressively upregulated pathways (Progressive I) related to energy production, metabolism, growth factors and glucose regulation. The interaction information about the genes was obtained from public interaction databases or the commercial Ingenuity package. In a network each node represents a gene and an edge represents an interaction (e.g. protein-protein, protein-DNA or protein-RNA). The nodes with high degree of connectivity (Top 20) are highlighted in yellow color. (PDF) [file pone.0062817.s003.pdf]

**A.**

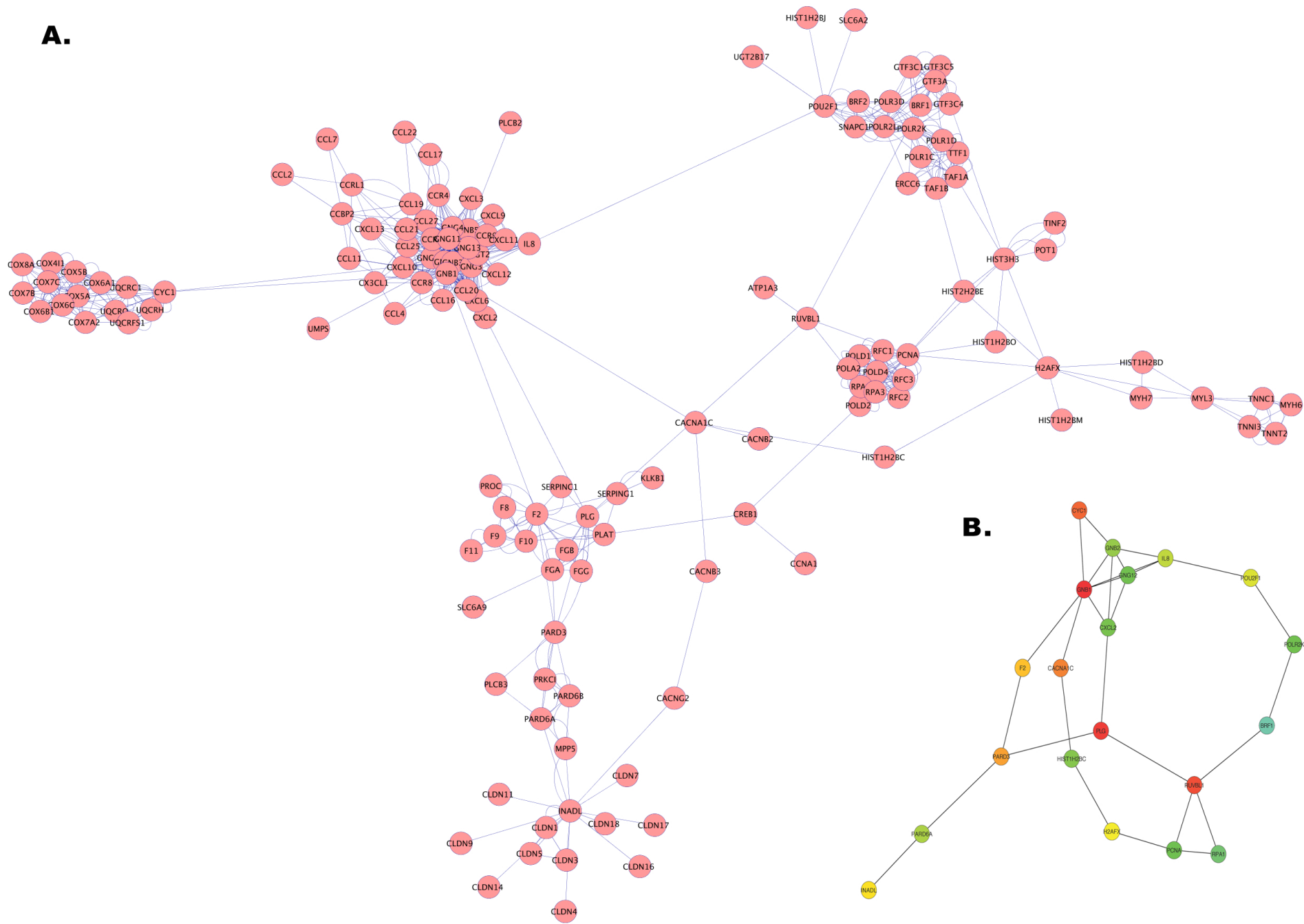

**B.**

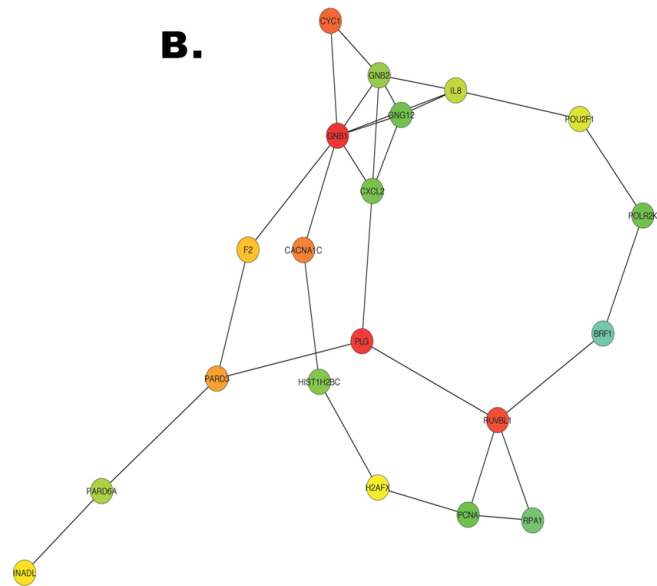

Supplement: Figure S4 — Interactive network and focus hubs of genes depicting Longterm Upregulation patterns. A) Interactive network, B) Top 20 focus genes. The interactive network and focus hub identification analysis was performed on genes from 14 Long-term Upregulated pathways linked to DNA stability, recombination and repair. In the network each node represents a gene and an edge represents an interaction. The focus gene hubs were identified using the bottleneck algorithm for identification of the most interactive molecules with a tree like topological structure. The bottleneck algorithm ranks genes on the basis of significance level with smaller rank indicating increasing confidence. The pseudocolor scale from red to green represents the bottleneck ranks from 1 to 20 (Fig. S4B). (PDF) [file pone.0062817.s004.pdf]

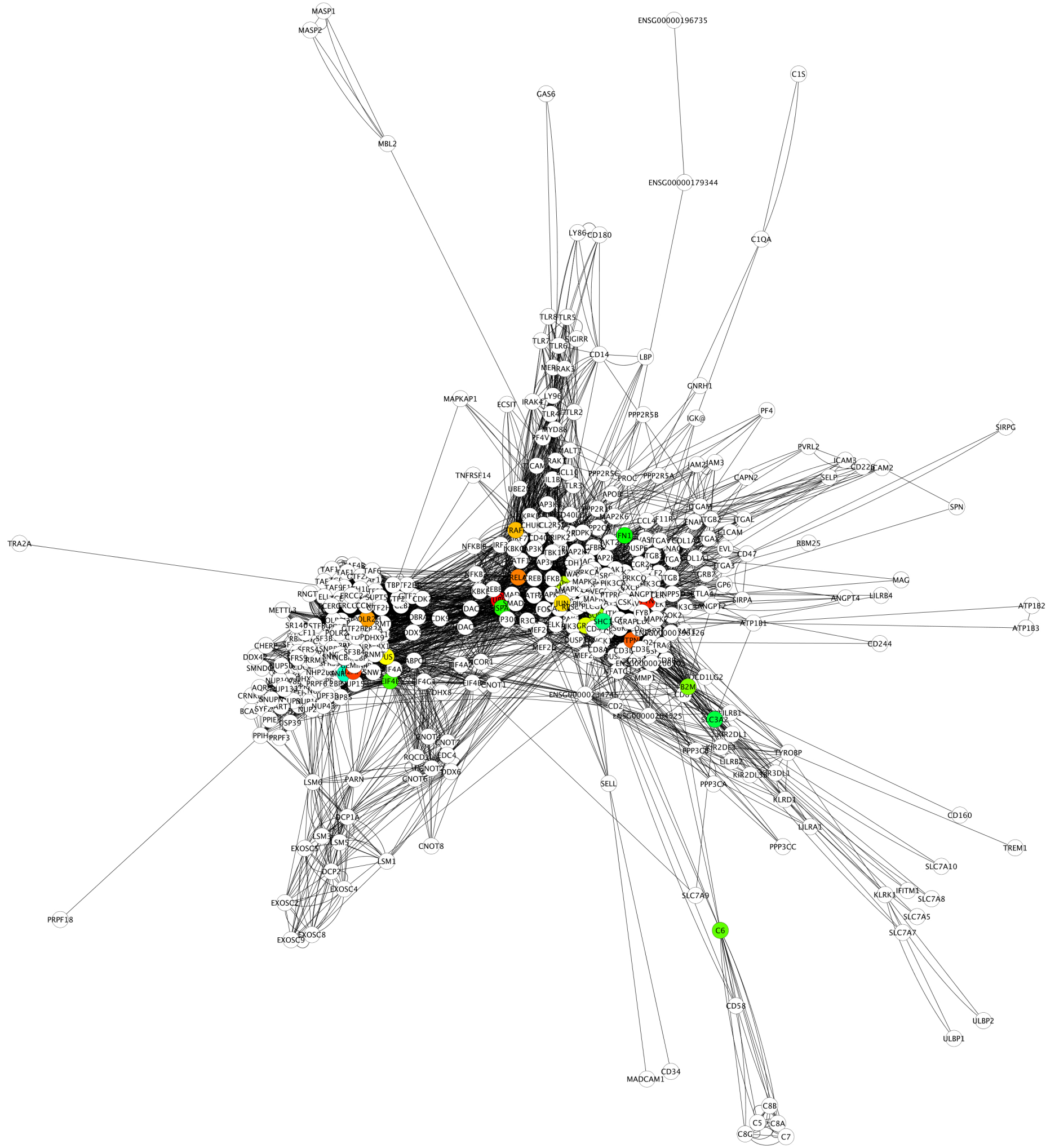

Supplement: Figure S5 — Interactive network and focus hubs of genes depicting acute Progressive (Progressive II) Downregulation patterns. The interactive network and focus hub identification analysis was performed on genes from 15 Progressively Downregulated (Progressive I) pathways linked to mRNA processing and immune response. The focus gene hubs were identified using the bottleneck algorithm for identification of the most interactive molecules with a tree like topological structure. The bottleneck algorithm ranks genes on the basis of significance level with smaller rank indicating increasing confidence. The pseudocolor scale from red to green represent bottleneck ranks from 1 to 20. (PDF) [file pone.0062817.s005.pdf]

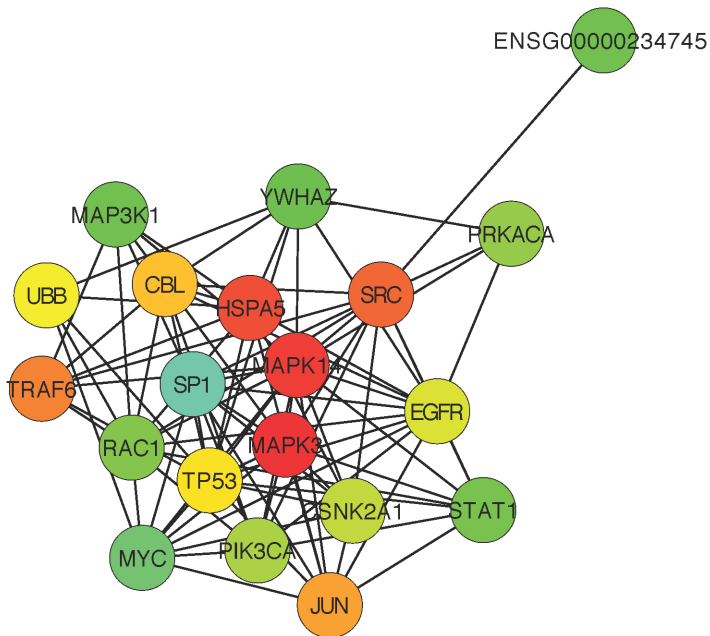

Supplement: Figure S6 — Top focus gene hubs identified from Interactive networks of significantly affected Long-term Downregulated pathways. The figure represents the top 20 focus genes identified from complex interactive networks generated from pathways with Long-term Downregulated patterns. The focus gene hubs were identified and ranked using the bottleneck algorithm for identification of the most interactive molecules with a tree like topological structure. The pseudocolor scale from red to green represent bottleneck ranks from 1 to 20 (smaller rank indicating increasing confidence). (PDF) [file pone.0062817.s006.pdf]
