# Supplementary material for: Relaxation Response Induces Temporal Transcriptome Changes in Energy Metabolism, Insulin Secretion and Inflammatory Pathways
Source: PLoS One. 2013 May 1;8(5):e62817. doi: 10.1371/journal.pone.0062817 (PMC3641112; doi:10.1371/journal.pone.0062817)
Supplement: Table S1 — Gene—ontology enrichment analysis of progressive and long—term expression patterns. (PDF) [file pone.0062817.s007.pdf]

Table S1: Gene-ontology enrichment analysis of progressive and long-term expression patterns.

**Progressive Upregulated**	**Enrichment Score**	**Related Processes**
System Development	10.6	Nervous System Development
Cell Differentiation	5.9	Regulation Of Cell Proliferation
Protein Binding	5.7	
Transmission Of Nerve Impulse	5.2	
Cell Adhesion And Communication	4.46	
Regulation Of Transcription From RNA Polymerase II Promote	4.2	
Muscle Contraction	4.05	
Blood Vessel Morphogenesis	3.8	Vasculature Development
Steroid Hormone Receptor Activity	3.5	
Gland Development	3.72	
Regulation Of Transport	3.36	
Positive Regulation Of Transcription, DNA-Dependent	3.25	Regulation Of Transcription From RNA Polymerase II Promoter
Glial Cell Differentiation	3.06	
Regulation Of Growth	3	
Response To Steroid Hormone Stimulus	2.8	
Positive Regulation Of Phospholipase C Activity	2.8	
Response To Vitamin	2.77	
Second-Messenger-Mediated Signaling	2.7	Regulation Of Camp Metabolic Process
Regulation Of Systemic Arterial Blood Pressure	2.4	
Oxidoreductase Activity	2.23	
Response To Oxygen Levels	2.23	
**Progressive Downregulated**		
mrna Processing	2.14	
Intracellular Protein Transport	2	
Antigen Processing And Presentation Of Peptide Or Polysaccharide Antigen Via MHC Class II	1.6	
Immune System Development	1.5	
Primary Metabolic Process	1.5	
Regulation Of DNA Binding	1.5	
Apoptosis	1.36	

**Long-Term Upregulated**		
Protein Binding	5.7	
Glutamine Family Amino Acid Catabolic Process	2.6	
Cellular Component Organization	2.6	
Cell-Matrix Adhesion	2.26	
Transcription Regulator Activity	2.15	
Regulation Of Neuronal Synaptic Plasticity	2.15	
Multicellular Organismal Response To Stress	2	
Cell Communication	2	
Cell Death	1.96	
Muscle Cell Differentiation	1.94	
Regulation Of Cytoskeleton Organization	1.79	
Nervous System Development	1.77	
Membrane Organization	1.67	
Transcription Repressor Activity	1.6	
Amine Transport	1.54	
ATPase Activity	1.53	
Ubiquitin Cycle	1.51	
**Long-Term Downregulated**		
Protein Binding	11.3	
Regulation Of Apoptosis	3.8	Cell Death, Apoptosis
Nuclear Transport	3.6	Protein, Macromolecule Transport
mrna Processing	2.8	
Cellular Metabolic Process	2.7	
Positive Regulation Of Cellular Process	2.4	
JAK-STAT Cascade	2.3	
T Cell Activation	2.1	Lymphocyte Activation,Leukocyte Activation,Immune System Process
Response To Insulin Stimulus	2.1	Regulation Of Glucose Transport
Cell Cycle	2	Cell Division,Cell Cycle Process,M Phase Of Mitotic Cell Cycle
Regulation Of DNA Binding	2	Negative Regulation Of NF-Kappab Transcription Factor Activity
Response To Stress	2	
