# Supplementary material for: Relaxation Response Induces Temporal Transcriptome Changes in Energy Metabolism, Insulin Secretion and Inflammatory Pathways
Source: PLoS One. 2013 May 1;8(5):e62817. doi: 10.1371/journal.pone.0062817 (PMC3641112; doi:10.1371/journal.pone.0062817)
Supplement: Table S2 — FeNO levels during one session of RR elicitation. (PDF) [file pone.0062817.s008.pdf]

Groups	ENO Levels		
	T1-T0	T2-T1	T2-T0
	Mean (SE)	Mean (SE)	Mean (SE)
M	1.93** (0.58)	0.07 (0.56)	2.18** (0.74)
N2	3.24*** (0.84)	-3.15*** (0.71)	-0.41 (0.72)
N1	1.52 (0.79)	-1.36** (0.49)	0.11 (0.89)

P value of paired-test for comparing ENO level between two time points within each study group: ** P <0.01; *** P <0.001
