# Supplementary material for: Relaxation Response Induces Temporal Transcriptome Changes in Energy Metabolism, Insulin Secretion and Inflammatory Pathways
Source: PLoS One. 2013 May 1;8(5):e62817. doi: 10.1371/journal.pone.0062817 (PMC3641112; doi:10.1371/journal.pone.0062817)
Supplement: Table S3 — Correlation analysis of NO levels and Selected 10 pathways affected progressively or only in long term manner by RR (Bold). The correlation analysis was performed both by comparing FeNO and gene expression levels at particular time point (e.g. T0, T1, T2) as well as changes in gene expression and FeNO levels within a group. The significance of the correlation was determined on the basis of P value (P<0.05) and FDR (<25%). The positive and negative correlations between FeNO and gene expression levels are indicated by red and green color respectively. (PDF) [file pone.0062817.s009.pdf]

Table S3: Correlation analysis of NO levels and Selected 10 pathways affected progressively or only in long term manner by RR (Bold).

The correlation analysis was performed both by comparing FeNO and gene expression levels at particular time point (e.g. T0, T1, T2) as well as changes in gene expression and FeNO levels within a group. The significance of the correlation was determined on the basis of P value (P < 0.05) and FDR (<25%). The positive and negative correlations between FeNO and gene expression levels are indicated by red and green color respectively.

Pathways	M group							N2 group						N1 group					
	T0	T1	T2	T2-T0		T2-T1		T2-T0		T2-T1		T1-T0		T2-T0		T2-T1		T1-T0	
KEGG_UBIQUITIN_MEDIATED_PROTEOLYSIS																			
KEGG_MTOR_SIGNALING_PATHWAY				0.05	0.17			0.004	0.01					0.01	0.18				
KEGG_CARDIAC_MUSCLE_CONTRACTION																			
KEGG_ANTIGEN_PROCESSING_AND_PRESENTATION	0.04					0.02	0.04												
BIOCARTA_RELA_PATHWAY			0			0.02	0.08			0.04	0.1								
BIOCARTA_IL7_PATHWAY				0.004	0.004														
BIOCARTA_NFKB_PATHWAY																			
BIOCARTA_MITOCHONDRIA_PATHWAY																			
BIOCARTA_STRESS_PATHWAY			0							0.004	0.09	0.01	0.12						
BIOCARTA_TNFR2_PATHWAY																0.01	0.12	0.04	0.19
	P value			Pvalue	FDR	Pvalue	FDR	Pvalue	FDR	Pvalue	FDR	Pvalue	FDR	Pvalue	FDR	Pvalue	FDR	Pvalu	FDR
