# Supplementary material for: Relaxation Response Induces Temporal Transcriptome Changes in Energy Metabolism, Insulin Secretion and Inflammatory Pathways
Source: PLoS One. 2013 May 1;8(5):e62817. doi: 10.1371/journal.pone.0062817 (PMC3641112; doi:10.1371/journal.pone.0062817)
Supplement: Text S1 — Supporting Information (PDF) [file pone.0062817.s010.pdf]

Supplementary Information

Relaxation Response induces temporal transcriptome changes in energy metabolism, insulin secretion and inflammatory pathways

Manoj K. Bhasin[1,4,5]*, Jeffery A. Dusek[6]*, Bei-Hung Chang[7,8]*, Marie G. Joseph[5], John W. Denninger[1,2], Gregory L. Fricchione[1,2], Herbert Benson[1,3†], Towia A. Libermann[1,4, 5†]

1- Benson-Henry Institute for Mind Body Medicine at Massachusetts General Hospital, Boston, MA 02114, USA

2- Department of Psychiatry, Massachusetts General Hospital, Harvard Medical School, Boston, MA 02114, USA

3- Department of Medicine, Massachusetts General Hospital, Harvard Medical School, Boston, MA 02114, USA

4- Department of Medicine, Division of Interdisciplinary Medicine and Biotechnology, Beth Israel Deaconess Medical Center, Harvard Medical School, Boston, MA 02115, USA

5- BIDMC Genomics and Proteomics Center, Beth Israel Deaconess Medical Center, Boston, MA 02115, USA

6- Institute for Health and Healing, Abbott Northwestern Hospital, Minneapolis, MN 55407, USA

7- VA Boston Healthcare System, Boston, MA

8- Department of Health Policy and Management, Boston University School of Public Health, Boston MA 02130, USA

A. Methods:

A.1 Samples:

Twenty-six healthy practitioners of various RR-eliciting techniques (including several types of meditation, Yoga, and repetitive prayer) participated in the time series study. Twenty-seven individuals without any prior RR-eliciting experience served as controls (N group; N1 =27). Novice subjects had 8-weeks of RR training, listened to a 20-minute RR-eliciting CD daily and are classified as N2 after 8 week of training (N2 =26). Whole blood (5 ml) was collected at 0, 20 and 35 minutes each of the study participants (Fig. 1). Total RNA extraction and purification was performed using previously described protocols (*1*).

A.2 Transcriptional profiling:

For transcriptional profiling, the Affymetrix human genome high throughput arrays plates with 96 arrays (HT U133A), containing more than 22,000 transcripts, was used. Microarray analysis was conducted by the BIDMC Genomics and Proteomics Center at the Beth Israel Deaconess Medical Center according to previously described protocols for total RNA extraction and purification, complementary DNA (cDNA) synthesis, *in vitro* transcription for production of biotin-labeled cRNA, hybridization of cRNA with human genome HT U133A Affymetrix plates, and scanning of image output file. The quality of scanned arrays images were determined on the basis of background values, percent present calls, scaling factors, and 3'-5' ratio of actin and GAPDH using the SimpleAffy package for R (*2*).

Scanned array images were analyzed by dChip (*3*). The raw probe level data was normalized using smoothing-spline invariant set method, and the signal value for each transcript was summarized using the PM-only based signal modeling algorithm in which the signal value corresponds to the absolute level of expression of a transcript (*3*). To calculate model based expression signal values, array and probe outliers were interrogated and image spikes were treated as signal outliers. The outlier detection was carried out using the dChip outlier detection algorithm. A chip was considered to be an outlier if the probe, signal or array outlier percentage exceeded a threshold of 10%. No chips were found to be outliers.

A.3 Gene Set Enrichment Analysis:

Gene Set Enrichment Analysis (GSEA) was used to determine whether an a priori defined sets of genes showed statistically significant, concordant differences between 2 groups (N2 vs. N1, and M vs. N1) or two time points (15 minutes vs. 35 minutes, 15 minutes vs. 50 minutes) in the context of known biological sets. GSEA is more powerful than conventional single-gene methods for studying the effects of interventions such as RR in which many genes each make subtle contributions.

GSEA calculates an enrichment score using the Kolmogorov-Smirnov test (KS-test) for determining whether a rank-ordered list of genes for a particular comparison of interest is enriched in a biologically related geneset. We have performed the enrichment analysis using the 880 canonical pathways derived from MSigDB2.0 (*4, 5*). The enriched gene sets have nominal p-value (NPV) less

than 5% and a False Discovery Rate (FDR) less than 25% after 500 random permutations. These criteria ensure that there is minimal chance of identifying false positives.

The genes from enriched pathways were merged into functional modules on the basis of overlap of significantly enriched genes using enrichment map plugin (*6*) in cytoscape: An Open Source Platform for Complex Network Analysis and Visualization (*7*). Genes with significant overlap (70% common genes) were considered neighbor and substitutable with each other. The patterns in significantly enrichment genesets from different comparisons (e.g. N vs. M, N1 vs. N2, 15 min vs. 35 min, 15 min vs. 50 min) were identified by developing a dotplots in lattice package. The interesting selected patterns were divided into the following major groups: i) long-term effects (M vs. N_1 or M vs. N_2), ii) progressive effects (changes which occur in both M and N_2 compared to N_1, and are of greater significance in M). Furthermore, by virtue of having three time points, we are able to describe constitutive changes (present at all three time points), acute changes (only present immediately after RR-elicitation) and delayed changes (only present 15 minutes after RR completion). Please see details for pattern classification in supplementary document.

A.4 Classification of GSEA enrichment patterns:

The patterns are explained in detail below:

i) <u>*Progressive I patterns*</u>: The Progressive-I Upregulated pattern consisted of gene sets that were significantly enriched in both N2 and M as compared to N1 and with greater enrichments in M (i.e., more

time points with significant enrichments in M compared to N1 and N2) (Fig. 2A, solid dots indicating significant group differences). In addition to these across group differences at each time point, most gene sets also showed significant changes across time points within each group (Fig. 2A, asterisks indicating significant time difference). Similarly, GSEA analysis identified Progressive-I Downregulated gene sets based on both across and within group comparisons.

ii) *Progressive II patterns*: GSEA identified pathways that depicted similar enrichments for M and N2 as compared to N1 at T1 and T2 in across group comparison. In addition to these across group differences at each time point, most gene sets also showed significant changes across time points within M group only. We classified these pathways as 'Progressive II' gene sets since the rapid enrichment within one session of RR practice can be evoked by short-term as well as long-term practitioners. In addition to across group changes, the M group is also able to depict the temporal changes in gene expression.

iii) *Long-term patterns:* GSEA identified pathways that were significantly upregulated in M at 2 or 3 time points compared to both N1 and N2, of which there were no significant group differences. In addition to these across group differences at each time point, most gene sets also showed significant changes across time points within M group only.

A.5 Self Organizing Map analysis:

To identify time and group dependent patterns from differentially expressed genes, we have adopted the Self Organizing Map (SOM) clustering technique(*8*). SOM allow the grouping of gene expression patterns into an imposed structure in which adjacent clusters are related, thereby identifying sets of genes that follow certain expression patterns across different conditions(*8*). We performed SOM clustering on transcript expression values using Pearson correlation coefficient based distance metrics and a target of 18 groups.

A.6 Gene Ontology (GO) enrichment analysis:

To identify the over-represented GO categories in the different gene expression patterns obtained from SOM clustering, we used the Biological Processes and Molecular Functions Enrichment Analysis available from the Database for Annotation, Visualization and Integrated Discovery (DAVID) (*9*). DAVID is an online implementation of EASE software that produces a list of over-represented categories using jackknife iterative resampling of the Fisher exact probabilities. A *p-value* gets assigned to each category on the basis of enrichments. Smaller *p-value* reflects increasing confidence in over-representation. The GO categories with *p-value* <0.05 were considered significant.

A.7 Pathways and Interactive network analysis:

We analyzed interactive networks and pathways for different patterns identified using SOM analysis of differentially expressed genes using the

commercial system biology oriented package Ingenuity Pathways Analysis (IPA 4.0) (http://www.ingenuity.com/). The knowledge base of this software consists of ontology and network models derived by systematically exploring the peer reviewed scientific literature. It calculates the P value using Fisher Exact test for each network and pathway according to the fit of user's data to the IPA database (*10*).(*10*). It displays the results as a score (-log *P value*) indicating the likelihood of a gene to be found in a network or pathways by random chance. For example, a network achieving a score of 2 has at least 99% confidence of not being generated by chance alone.

B. References:

1. J. A. Dusek, H. H. Otu, A. L. Wohlhueter, M. Bhasin, L. F. Zerbini, M. G. Joseph *et al.*, Genomic counter-stress changes induced by the relaxation response. *PLoS One* 2008; 3: e2576.

2. C. L. Wilson, C. J. Miller, Simpleaffy: a BioConductor package for Affymetrix Quality Control and data analysis. *Bioinformatics* 2005; 21: 3683-3685.

3. C. Li, W. H. Wong, Model-based analysis of oligonucleotide arrays: expression index computation and outlier detection. *Proc Natl Acad Sci U S A* 2001; 98: 31-36.

4. A. Subramanian, H. Kuehn, J. Gould, P. Tamayo, J. P. Mesirov, GSEA-P: a desktop application for Gene Set Enrichment Analysis. *Bioinformatics* 2007; 23: 3251-3253.

5. A. Subramanian, P. Tamayo, V. K. Mootha, S. Mukherjee, B. L. Ebert, M. A. Gillette *et al.*, Gene set enrichment analysis: a knowledge-based approach for interpreting genome-wide expression profiles. *Proc Natl Acad Sci U S A* 2005; 102: 15545-15550.

6. D. Merico, R. Isserlin, O. Stueker, A. Emili, G. D. Bader, Enrichment map: a network-based method for gene-set enrichment visualization and interpretation. *PLoS One* 2010; 5: e13984.

7. P. Shannon, A. Markiel, O. Ozier, N. S. Baliga, J. T. Wang, D. Ramage *et al.*, Cytoscape: a software environment for integrated models of biomolecular interaction networks. *Genome Res* 2003; 13: 2498-2504.

8. P. Tamayo, D. Slonim, J. Mesirov, Q. Zhu, S. Kitareewan, E. Dmitrovsky *et al.*, Interpreting patterns of gene expression with self-organizing maps: methods and application to hematopoietic differentiation. *Proc Natl Acad Sci U S A* 1999; 96: 2907-2912.

9. W. Huang da, B. T. Sherman, Q. Tan, J. Kir, D. Liu, D. Bryant *et al.*, DAVID Bioinformatics Resources: expanded annotation database and novel algorithms to better extract biology from large gene lists. *Nucleic Acids Res* 2007; 35: W169-175.

10. R. Fisher, On the Interpretation of x^2 from Contingency Tables, and the Calculation of P. *Journal of the Royal Statistical Society* 1922; 85: 7.

C. Supporting Information Legends:

Figure S1: Schematic view of temporal relaxation response study design and analysis plans. The transcriptome profiling was performed on peripheral blood mononuclear cells (PBMCs) collected immediately prior to (T0), immediately after (T1) and 15 minutes after (T2) listening to a 20-minute Education CD by the Novices (N1) or a 20-minute RR CD by the Short term practitioners (N2) and the Long term practitioners (M). The global transcriptome of PBMCs was profiled using HT_U133A arrays containing >22,000 transcripts. The transcriptome data were analyzed using high-level bioinformatics algorithms to identify differentially expressed transcripts, significantly affected pathways and systems biology networks that are related to RR elicitation. The expression patterns were generated from differentially expressed genes using Self-Organizing Maps (SOM) analysis. The results of the GSEA from all comparisons were classified to temporal patterns (e.g. Progressive, Long) by developing a R-language script.

Figure S2: Temporal genomic expression patterns during one session of RR elicitation. Genes that were differentially expressed either across or within groups comparisons at different time point were used as seed sets of genes for Self-Organizing Map (SOM) analysis. These differentially expressed genes were partitioned to 18 separate maps according to Pearson correlation coefficient based distance metrics. Each pattern represents a set of genes that depict a similar expression pattern suggesting that they are biologically linked to a specific

function. The figure displays the box plot of the gene expression with X-axis representing time points and groups, and Y-axis representing scaled gene expression data from -1 to +1. The patterns are merged into 10 expression categories on the basis of similarities in expression patterns.

Figure S3: Interactive Network of progressively (Progressive II) upregulated genes. The network was generated from genes of 27 progressively upregulated pathways (Progressive I) related to energy production, metabolism, growth factors and glucose regulation. The interaction information about the genes was obtained from public interaction databases or the commercial Ingenuity package. In a network each node represents a gene and an edge represents an interaction (e.g. protein-protein, protein-DNA or protein-RNA). The nodes with high degree of connectivity (Top 20) are highlighted in yellow color.

Figure S4: Interactive network and focus hubs of genes depicting Long-term Upregulation patterns. A) Interactive network, B) Top 20 focus genes. The interactive network and focus hub identification analysis was performed on genes from 14 Long-term Upregulated pathways linked to DNA stability, recombination and repair. In the network each node represents a gene and an edge represents an interaction. The focus gene hubs were identified using the bottleneck algorithm for identification of the most interactive molecules with a tree like topological structure. The bottleneck algorithm ranks genes on the basis of significance level with smaller rank indicating increasing confidence. The

pseudocolor scale from red to green represents the bottleneck ranks from 1 to 20 (Fig S4B).

Figure S5: Interactive network and focus hubs of genes depicting acute Progressive (Progressive II) Downregulation patterns. The interactive network and focus hub identification analysis was performed on genes from 15 Progressively Downregulated (Progressive I) pathways linked to mRNA processing and immune response. The focus gene hubs were identified using the bottleneck algorithm for identification of the most interactive molecules with a tree like topological structure. The bottleneck algorithm ranks genes on the basis of significance level with smaller rank indicating increasing confidence. The pseudocolor scale from red to green represent bottleneck ranks from 1 to 20.

Figure S6: Top focus gene hubs identified from Interactive networks of significantly affected Long-term Downregulated pathways. The figure represents the top 20 focus genes identified from complex interactive networks generated from pathways with Long-term Downregulated patterns. The focus gene hubs were identified and ranked using the bottleneck algorithm for identification of the most interactive molecules with a tree like topological structure. The pseudocolor scale from red to green represent bottleneck ranks from 1 to 20 (smaller rank indicating increasing confidence).
